# Carpal Tunnel Syndrome: Pathophysiology and Comprehensive Guidelines for Clinical Evaluation and Treatment

**DOI:** 10.7759/cureus.27053

**Published:** 2022-07-20

**Authors:** Aditya Joshi, Karan Patel, Aleem Mohamed, Solomon Oak, Michelle H Zhang, Hailey Hsiung, Alex Zhang, Urvish K Patel

**Affiliations:** 1 Medical School, Cooper Medical School of Rowan University, Camden, USA; 2 Orthopaedics, Cooper Medical School of Rowan University, Camden, USA; 3 Psychological & Brain Sciences and Biology, Johns Hopkins University, Baltimore, USA; 4 Healthcare (Social Sciences), Independent, Highland Park, USA; 5 Public Health and Neurology, Icahn School of Medicine at Mount Sinai, New York, USA

**Keywords:** carpal tunnel syndrome, art of diagnosis, comprehensive physical exam, review article, treatment guidelines

## Abstract

In carpal tunnel syndrome (CTS), the median nerve is compressed at the level of the carpal tunnel in the wrist. This entrapment manifests as unpleasant symptoms, such as burning, tingling, or numbness in the palm that extends to the fingers. As the disease progresses, afflicted individuals also report decreased grip strength accompanied by hand weakness and restricted movement. The first half of this review elaborates on CTS pathology by providing readers with a comprehensive understanding of the etiology, relevant anatomy, and disease mechanism.

CTS is considered the most common entrapment neuropathy, affecting around 3-6% of the adult population. Further, CTS prevalence has seen a dramatic increase in the last few decades paralleling the growth of everyday technology usage. Despite how common it is to have CTS, it can be quite challenging for physicians to make a definite diagnosis due to differentials that present with overlapping symptoms. Even more difficult can be deciding on a course of treatment that is the most effective and considerate of patient needs. Thus arises the need for clear clinical direction, and hence we end with a discussion around such guidelines that serve as a starting point toward effective diagnoses and patient treatment.

## Introduction and background

Carpal tunnel syndrome (CTS) is widely regarded as the most common compressive neuropathy. In the United States, an incidence of 1-3 per 1,000 people and a prevalence of 50 per 1,000 individuals have been reported [1]. The peak incidence years are between the ages of 45 and 54 [2], and women are three times more likely to be afflicted by CTS than males [3]. CTS is often listed as an occupational hazard, with the US Department of Labor and Statistics reporting the occupational incidence rate of CTS to be 0.5 per 10,000 workers [2], costing employers billions in workers’ compensation. A report by the Centers for Disease Control and Prevention (CDC) identified the most at-risk occupations to be production workers, material movers, and office administrative staff [4]. These occupations require repetitive motion with hands. Occupational cases of CTS have an average duration of 27 days away from work, and nearly 23% of workers may not return to their previous work even after undergoing surgical treatment [5].

## Review

Relevant anatomy

To fully understand the carpal tunnel, one must first begin with learning the basic anatomy of the forearm and the nerves that are associated with it. While complex, the relevant jargon serves as a prerequisite to understanding this syndrome. In the following sections, we detail the relevant anatomy such that readers can obtain a more complete appreciation for CTS starting from the fundamentals.

Anatomy of the carpal tunnel

Before the median nerve can make its way to the wrist, it passes through the carpal tunnel [6]. The carpal tunnel is an anatomical landmark whose depression is made up of the proximal (scaphoid, trapezium, lunate, pisiform) and the distal row (trapezium, trapezoid, capitate, and hamate) [7,8] of carpal bones. The roof of the tunnel is called the flexor retinaculum, and it attaches to the scaphoid and trapezium on the lateral side of the wrist and the hamate and pisiform on the medial side [9]. The carpal tunnel serves as a passageway for the extrinsic tendons of the forearm and prevents them from bowing as the wrist is flexed. The extrinsic tendons of the forearm that run through the carpal tunnel are the four flexor digitorum superficialis tendons, the four flexor digitorum profundus tendons, and the flexor pollicis longus tendon [10].

Pathophysiology of carpal tunnel syndrome

**Figure 1:**
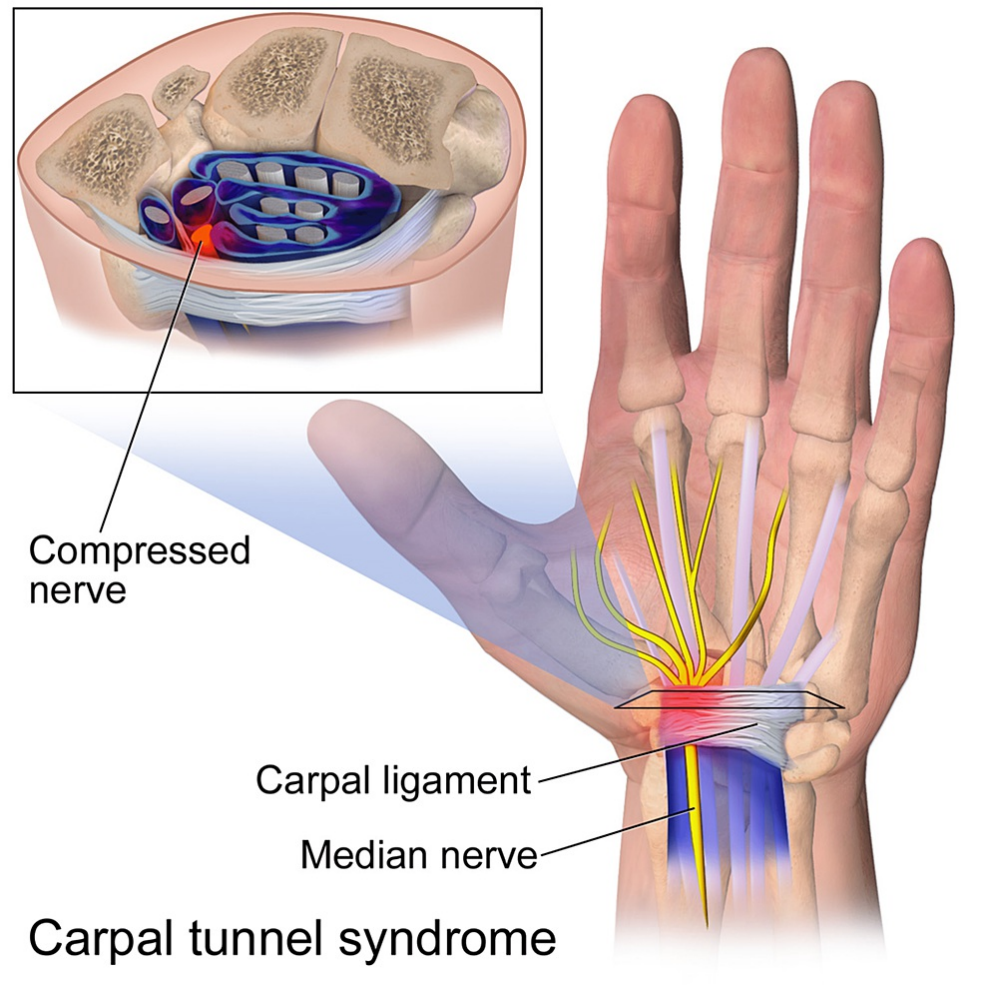
Pathophysiology of carpal tunnel syndrome illustrating the compression of the median nerve within the carpal tunnel space. This image is available under the Creative Commons CC-BY-NC license and permits non-commercial use, distribution, and reproduction in any medium provided the original work is properly cited [11].

While most cases of CTS are idiopathic, the pathophysiology of CTS can be simplified to compression of the median nerve at the carpal tunnel level, as illustrated in Figure 1 [11]. There are multiple mechanisms that can lead to median nerve entrapment along this passageway. The two major sites of compression are at the outlet of the tunnel under the flexor retinaculum roof and at the hamulus of the hamate. Compression can arise from increased compartmental pressure in the carpal tunnel, and the most common mechanism of this is hypertrophy of the synovial tissue that surrounds the extrinsic tendons of the forearm. This hypertrophy is an inflammatory response to extensive use, trauma to the wrist, or an underlying inflammatory process such as arthritis.

Activities that excessively engage wrist flexion or prolonged wrist movements have been noted to increase the fluid pressure [12] and result in ischemic injury to the median nerve. There are several plausible mechanisms that can similarly lead to ischemic injuries, such as the breakdown of the blood-nerve barrier producing a microcompartment syndrome in the carpal tunnel, fibrotic thickening of the vasculature, or a dysfunction of the microvasculature resulting in intraneural edema [13]. Compression can also result from obstruction in the carpal tunnel following trauma to the wrist joint.

A high-energy trauma in young patients or low-energy trauma in elderly patients can result in volar displacement of the lunate bone into the carpal tunnel, resulting in gross obstruction. Common causes of injury include falling onto an outstretched hand or motor vehicle accidents. Although relatively uncommon, this displacement can lead to entrapment of the median nerve [14].

While it is critical to explore the mechanisms behind the common, easily discernible CTS pathophysiology, it is equally important to discuss the less-apparent exacerbated night pains associated with CTS. Many individuals report an increase in CTS symptoms at night during periods of inactivity. This is due to a couple of reasons; individuals resting in the supine position have redistribution of fluid into their distal limbs which increases the pressure. There is also a lack of interstitial drainage in this position due to the lack of muscle pump and movement, further contributing to the increased pressure due to lack of clearance. Individuals often flex their wrists at night, and the prolonged flexion further compresses the roof of the tunnel against the median nerve [15].

Understanding the common signs and risk factors

Understanding the mechanism behind carpal tunnel also allows us to understand why certain actions and risk factors may lead to the development of, or even episodic worsening of, the syndrome. Common signs associated with carpal tunnel include aggravation of symptoms at night, and while driving, on the phone, or typing on a computer keyboard. As one may have guessed, most of these scenarios (and similar ones that are CTS-associated) involve the individual flexing their wrists, which further compresses the carpal tunnel onto itself leading to an increase in pressure to the area. Another common sign of CTS is paresthesias in the thumb, index, middle, and medial half of the ring finger on the palmar surface of the hand. This finding can also be explained by an understanding of the anatomy. After the median nerve crosses the carpal tunnel in the hand, it supplies the sensory innervation to these digits. Naturally, compression of the nerve explains the burning/tingling sensation in the sensory distribution of the nerve that many afflicted individuals complain of [16].

Common risk factors for CTS include, but are not limited to, gender, inflammatory conditions, pregnancy, diabetes, and hypertension. In the following, we explain these risk factors based on the current understanding of the pathophysiology associated with CTS.

● With respect to gender, the female predominance of the condition is hypothesized to stem from the fact that females tend to have a smaller relative cross-sectional area of the carpal tunnel than males at 9.0 and 11.3, respectively [17]. Therefore, females may be more susceptible to compression of the median nerve within this area. However, from an occupational viewpoint, this predominance may be in part due to females being overrepresented in occupations or assigned to tasks with a higher risk of CTS [18].

● Inflammatory conditions, such as rheumatoid arthritis (RA), lead to synovial hyperplasia (lining of the joints). The infiltrating pannus can narrow the space in the carpal tunnel and cause compression of the median nerve [19].

● Pregnancy is very similar to edema, in that during pregnancy the body tends to retain more fluid which may lead to an increase in pressure. Further, pregnancy has been shown to alter levels of hormones such as insulin; moreover, glucose metabolism has been associated with CTS development [20].

● With diabetes, the hyperglycemic conditions associated with the disease cause glycosylation and inflammation of the tendons which prevents them from gliding past one another as they normally do. Instead, these conditions promote stretching in the tendons [21].

● Finally, hypertension has been found to have an initial protective effect against CTS that deteriorates to a risk factor in the long term. Hypertension presents with an elevation in arterial pressure. Thus, the loss of blood flow from compression can be temporarily countered by hypertensive effects. However, this relief is short-lived because the long-term effects of hypertension can produce small-vessel disease. The vasculature will ultimately become compromised and the initial compensatory effects of hypertension will not suffice [22].

Ways to test for carpal tunnel syndrome

The approach to diagnosing carpal tunnel syndrome includes physical tests as well as diagnostic testing via imaging and electrophysiological measures. Physical examination techniques for CTS include Phalen’s and Durkan’s tests. Phalen’s test is a maneuver that requires a patient to push the dorsal surface of their hands together for 30-60 seconds. This technique increases the pressure on the median nerve by further compressing it between the flexor retinaculum and the distal end of the radius. The test is positive if the patient reproduces the symptoms associated with CTS, in particular, paresthesia. The sensitivity of the test is 0.5 and the specificity is 0.33. Durkan’s test is a more provocative procedure where a patient holds out their hand in a supinated position while an examiner places one thumb directly over the carpal tunnel along the course of the median nerve distal to the flexor retinaculum. The examiner then places their other thumb over the first and applies sustained pressure for 15-30 seconds. Similar to Phalen’s test, the patient is assessed for the reproduction of symptoms, namely, paresthesia. The sensitivity of the test is 0.77 and the specificity is 0.22 [23]. Upon physical examination, CTS also presents with a positive Tinel sign. A physician taps the median nerve proximal to the flexor retinaculum and observes if this elicits any numbness/tingling in the digits innervated by the median nerve [24]. However, physical examinations are not sufficient for a definitive diagnosis and are often used adjunctively with other diagnostic tests.

Electrophysiological studies can clarify any uncertainty regarding a CTS diagnosis. A nerve conduction study (NCS) tests the integrity of the median nerve by measuring the conduction velocity of the nerve across the carpal tunnel. The presence of slower conduction times or a delayed distal response by the median nerve innervations in digits two or three indicates a positive electrodiagnosis [25]. Electromyography (EMG) is an electrodiagnostic test for the integrity of the muscles innervated by the median nerve, namely, the abductor pollicis brevis. If there are any changes in the motor unit action potentials or a notable increase in spontaneous activity in the muscle, the EMG confirms a positive diagnosis [26]. In regards to diagnosis via imaging, the most common modality is ultrasound. The presentation of CTS on ultrasound is demonstrated by an increased cross-sectional area in the median nerve and outward flaring of the flexor retinaculum due to an enlarged median nerve [27]. Review of Class I and Class II evidence-based studies suggest ultrasound is accurate at diagnosing CTS and is recommended as a supplement to electrodiagnostic studies [28].

Treatment options

Treatment of CTS ranges from conservative techniques such as nightly bracing to more aggressive management such as surgical removal of the flexor retinaculum. The American Academy of Orthopedic Surgeons (AAOS) guidelines strongly recommend immobilizing the wrist to improve patient outcomes [29]. A wrist brace offers a simple solution to the compression along the median nerve by stabilizing the wrist joint in a position that is often neutral, but occasionally slightly extended, to mitigate the pressure exerted on the median nerve. Typically, the brace is worn at night but can also be recommended for activities that can exacerbate the symptoms of CTS, such as when typing on a computer/keyboard. However, because the splint somewhat immobilizes the joint, there is the possibility of muscle weakness in the wrist with prolonged bracing [30].

Other non-operative procedures also include non-steroidal anti-inflammatory drug (NSAID) use or corticosteroid injections at the carpal tunnel to reduce inflammation of the synovial tissue. AAOS guidelines highly recommend steroid injections and moderately recommend oral treatments such as NSAIDs [29]. NSAIDs inhibit prostaglandin synthesis which are the primary mediators of inflammation associated with CTS. The analgesic and anti-inflammatory properties of NSAIDs can provide short-term relief but are not curative. Corticosteroids exert anti-inflammatory effects by altering the genetic transcription of inflammatory genes. After translocating to a cell’s nucleus, the steroid binds to glucocorticoid responsive elements and forms a complex that can inhibit histone acetyltransferases or recruit histone deacetylases to reduce transcription of inflammatory genes or promoters of these genes. Corticosteroids can also conversely, instead of suppressing inflammatory genes, promote anti-inflammatory ones. Similar to NSAIDs, steroid injections can reduce the discomfort with CTS but symptoms can reappear in the long term [31].

Additional conservative approaches include hand therapy which aims to improve the gliding among the carpal tunnel ligaments and nerves. This therapy introduces more space in the carpal tunnel through gentle stretching. The increased mobilization improves nerve functionality while the additional space from stretching exercises reduces compartmental pressure [32]. Ultrasound has been found to have a diagnostic use as well as a therapeutic use for CTS. While the mechanism is not entirely elucidated, the delivery of high-frequency waves can produce anti-inflammatory effects and improve nerve conduction [33].

Ultimately, though, if a patient’s prognosis is severe, operative intervention may be necessary to treat their CTS. AAOS guidelines recommend surgery for individuals who have the risk of long-term complications, including, but not limited to, hand muscle atrophy, irreversible median nerve damage, and/or inability to continue their occupation. Prior to surgery, guidelines recommend electrodiagnostic studies to confirm the diagnosis [29]. For the surgical procedure, the flexor retinaculum is removed to relieve the pressure on the median nerve [34].

Importance of proper physician examination/physician-associated challenges

Because of the prevalence of CTS in the population, physicians need to be informed on the best clinical practice guidelines for its diagnosis and treatment. A comprehensive review of the literature recommends the following evidence-based guidelines: a detailed patient history with pertinent questions regarding CTS, a proper diagnostic physical examination, electrodiagnostic tests if necessary to confirm the differential, and, lastly, the optimal treatment given the severity of the condition [35]. It is important for such guidelines to be in place so that physicians can recognize symptoms and respond in a timely manner to prevent permanent median nerve damage. In the United States, the medical costs related to the diagnosis and treatment of CTS can be as high as 13,000 dollars per surgical case, upwards of 40,000 annually for nonoperative cases, [36] and as noted earlier have resulted in billions of dollars of expenditure for workers’ compensation. Thus, appropriate identification and management of CTS have fiscal merits. However, this examination hinges on physician capacity and does not come without challenges. If not presented with a proper set of guidelines or lacking technology such as electrodiagnostic tests to confirm the diagnosis, the condition can be difficult for physicians to navigate. With guidelines in place, physicians can reproduce diagnoses with high accuracy and have transparency with patients [37].

In addition to physician-associated challenges that can arise from logistical limitations, navigating CTS can be difficult because of a broad differential that can present with similar symptoms [38].

Several types of arthritis present with the same pain, numbness/tingling, and decrease in range of motion associated with CTS [39]. In contrast, arthritis presentation can include nodular growths or osteophytes in the affected joint that are not present in CTS, and while arthritis affects all digits, CTS does not affect the fifth digit [40].

A nerve root compression due to spinal disc herniation could also reproduce the paresthesias and decreased functionality found in CTS. As noted above, the brachial plexus provides innervation to the nerves running the length of the arm, including the median nerve. Radiculopathy of these nerve roots by a slipped disc, particularly C6-C7/C7-C8, will produce the symptoms found in the hand with CTS. However, because CTS is a distal neuropathy, its symptoms are limited to the distal limb whereas a disc herniation would follow the length of a dermatome from the trunk. Therefore, careful mapping of sensory deficits is imperative to rule out disc herniation.

Thoracic outlet syndrome is a neurovascular compression disorder that can increase pressure on the nerves and vessels found in the thoracic outlet [41]. This compression can be a result of the presence of a cervical rib, hypertrophy of the anterior scalene muscle, prominent C7 transverse processes infringing on the region, or a Pancoast tumor [42]. The compression of the brachial plexus can present with symptoms such as tingling/discomfort in the hand [43]. Rare instances of thoracic outlet syndrome can present with atrophy of the intrinsic muscles of the hand [44]. Similar to a spinal disc herniation, thoracic outlet syndrome is a proximal nerve impingement and presents with upper limb deficits in addition to the distal ones [45]. The possibility of thoracic outlet syndrome can be excluded without these symptoms as well as with a negative result on a maneuver known as the Adson’s test [46].

A common differential that also presents similarly to CTS is repetitive overuse resulting in a strain in the intrinsic muscles of the hand or tendonitis [47]. These conditions primarily affect the muscles rather than nerves. Patients present with myalgias and numbness/tingling in various regions of the hand that may overlap with the area affected by CTS [48].

## Conclusions

From this broad overview emerges the importance of comprehensive care in addition to ongoing research in regards to optimizing patient diagnosis and treatment. In the long run, efficient diagnosis and effective treatment result in improved quality of medical care and quality of life, as well as fiscal merits for both patients and healthcare/occupational systems. This is only possible with an extensive understanding of the pathophysiological causes behind median nerve compression and subsequent disease. For proper diagnosis, a thorough patient assessment is critical, and physicians should use a combination of patient history and physical examinations in conjunction with electrophysiological diagnostic tests, being careful to balance being meticulous without being excessive. Treatment plans should largely be personalized based on the underlying mechanism and severity of each case. In other words, while largely prevalent in the adult population, CTS care is not one-size-fits-all and often requires careful planning and critical thinking.

## References

[1] Sevy JO, Varacallo M (2022). Carpal tunnel syndrome. https://www.ncbi.nlm.nih.gov/books/NBK448179/.

[2] (2022). U.S. Department of Labor. Nonfatal occupational injuries and illnesses requiring days off from work. https://www.bls.gov/news.release/pdf/osh2.pdf.

[3] McDiarmid M, Oliver M, Ruser J, Gucer P (2000). Male and female rate differences in carpal tunnel syndrome injuries: personal attributes or job tasks?. Environ Res.

[4] Jackson R, Beckman J, Frederick M, Musolin K, Harrison R (2018). Rates of carpal tunnel syndrome in a state workers' compensation information system, by industry and occupation - California, 2007-2014. MMWR Morb Mortal Wkly Rep.

[5] (2022). Bureau of Labor Statistics, U.S. Department of Labor. The Economics Daily. Distribution of days away from work due to workplace injuries and illnesses. https://www.bls.gov/opub/ted/2007/nov/wk4/art02.htm.

[6] Robbins H (1963). Anatomical study of the median nerve in the carpal tunnel and etiologies of the carpal-tunnel syndrome. J Bone Joint Surg Am.

[7] Srinivas Reddy R, Compson J (2005). (i) Examination of the wrist—surface anatomy of the carpal bones. Curr Orthop.

[8] Tang A, Varacallo M (2022). Anatomy, shoulder and upper limb, hand carpal bones. https://www.ncbi.nlm.nih.gov/books/NBK535382/.

[9] Davies K (2022). TeachMeAnatomy. The carpal tunnel. https://teachmeanatomy.info/upper-limb/areas/carpal-tunnel/.

[10] Presazzi A, Bortolotto C, Zacchino M, Madonia L, Draghi F (2011). Carpal tunnel: normal anatomy, anatomical variants and ultrasound technique. J Ultrasound.

[11] Blausen.com staff (2014). Medical gallery of Blausen Medical 2014. WikiJournal of Med.

[12] Werner RA, Andary M (2002). Carpal tunnel syndrome: pathophysiology and clinical neurophysiology. Clin Neurophysiol.

[13] Aboonq MS (2015). Pathophysiology of carpal tunnel syndrome. Neurosciences (Riyadh).

[14] Bhatia M, Sharma A, Ravikumar R, Maurya VK (2017). Lunate dislocation causing median nerve entrapment. Med J Armed Forces India.

[15] Patel A, Culbertson MD, Patel A, Hashem J, Jacob J, Edelstein D, Choueka J (2014). The negative effect of carpal tunnel syndrome on sleep quality. Sleep Disord.

[16] Dydyk AM, Negrete G, Sarwan G (2022). Median nerve injury. https://www.ncbi.nlm.nih.gov/books/NBK553109/.

[17] Sassi SA, Giddins G (2016). Gender differences in carpal tunnel relative cross-sectional area: a possible causative factor in idiopathic carpal tunnel syndrome. J Hand Surg Eur Vol.

[18] Nakamichi K, Tachibana S (1998). Histology of the transverse carpal ligament and flexor tenosynovium in idiopathic carpal tunnel syndrome. J Hand Surg Am.

[19] Feldon P, Terrono AL (2006). Carpal tunnel syndrome in rheumatoid arthritis. Tech Orthop.

[20] Osterman M, Ilyas AM, Matzon JL (2012). Carpal tunnel syndrome in pregnancy. Orthop Clin North Am.

[21] Singh R, Gamble G, Cundy T (2005). Lifetime risk of symptomatic carpal tunnel syndrome in type 1 diabetes. Diabet Med.

[22] Guan W, Lao J, Gu Y, Zhao X, Rui J, Gao K (2018). Case-control study on individual risk factors of carpal tunnel syndrome. Exp Ther Med.

[23] Zhang D, Chruscielski CM, Blazar P, Earp BE (2020). Accuracy of provocative tests for carpal tunnel syndrome. J Hand Surg Glob Online.

[24] Ho T, Braza ME (2022). Hoffmann Tinel sign. https://pubmed.ncbi.nlm.nih.gov/32310394/.

[25] Alanazy MH (2017). Clinical and electrophysiological evaluation of carpal tunnel syndrome: approach and pitfalls. Neurosciences (Riyadh).

[26] Rosario NB, De Jesus O (2022). Electrodiagnostic evaluation of carpal tunnel syndrome. StatPearls [Internet].

[27] (2022). Radiopaedia.org. Carpal tunnel syndrome. https://radiopaedia.org/articles/carpal-tunnel-syndrome-1?lang=us#:~:text=the%20affected%20nerve.-,Ultrasound,distal%20flattening%20of%20the%20nerve.

[28] Cartwright MS, Hobson-Webb LD, Boon AJ (2012). Evidence-based guideline: neuromuscular ultrasound for the diagnosis of carpal tunnel syndrome. Muscle Nerve.

[29] (2022). American Academy of Orthopaedic Surgeons. Management of carpal tunnel syndrome evidence-based clinical practice guideline. https://www.aaos.org/globalassets/quality-and-practice-resources/carpal-tunnel/cts-cpg_4-25-19.pdf.

[30] Ostergaard PJ, Meyer MA, Earp BE (2020). Non-operative treatment of carpal tunnel syndrome. Curr Rev Musculoskelet Med.

[31] Barnes PJ (2006). How corticosteroids control inflammation: Quintiles Prize Lecture 2005. Br J Pharmacol.

[32] Ünver S, Akyolcu N (2018). The effect of hand exercise on reducing the symptoms in hemodialysis patients with carpal tunnel syndrome. Asian J Neurosurg.

[33] Page MJ, O'Connor D, Pitt V, Massy-Westropp N (2013). Therapeutic ultrasound for carpal tunnel syndrome. Cochrane Database Syst Rev.

[34] (2022). Carpal tunnel release. https://www.hopkinsmedicine.org/health/treatment-tests-and-therapies/carpal-tunnel-release.

[35] (2021). Carpal tunnel syndrome: clinical manifestations and diagnosis. https://www.uptodate.com/contents/carpal-tunnel-syndrome-clinical-manifestations-and-diagnosis.

[36] Gabrielli AS, Lesiak AC, Fowler JR (2020). The direct and indirect costs to society of carpal tunnel release. Hand (N Y).

[37] Keith MW, Masear V, Chung K (2009). Diagnosis of carpal tunnel syndrome. J Am Acad Orthop Surg.

[38] Lee MJ, LaStayo PC (2004). Pronator syndrome and other nerve compressions that mimic carpal tunnel syndrome. J Orthop Sports Phys Ther.

[39] Cleveland Clinic (2022). Cleveland Clinic. Carpal tunnel syndrome: risk factors, symptoms & treatment. Cleveland Clinic.

[40] Abhishek A, Doherty M (2013). Diagnosis and clinical presentation of osteoarthritis. Rheum Dis Clin North Am.

[41] Mayo Clinic (2022). Mayo Clinic: thoracic outlet syndrome. Mayo Clinic.

[42] Ebraheim Ebraheim, N N (2022). Medium. Thoracic outlet syndrome. https://medium.com/thrive-global/thoracic-outlet-syndrome-2f8840d0e438.

[43] Jones MR, Prabhakar A, Viswanath O (2019). Thoracic outlet syndrome: a comprehensive review of pathophysiology, diagnosis, and treatment. Pain Ther.

[44] (2022). Thoracic outlet syndrome. Johns Hopkins Medicine.

[45] Kaplan J, Kanwal A (2022). Thoracic outlet syndrome. https://www.ncbi.nlm.nih.gov/books/NBK557450/.

[46] (2022). Physiopedia contributors. Adsons test. Physiopedia.

[47] (2022). Penn Medicine. Tendonitis treatments. Pennmedicine.org.

[48] (2022). OrthoNY. Is it tendonitis or carpal tunnel syndrome?. OrthoNY.

